# Supplementary material for: Identification of Freezing-Responsive microRNAs and Their Targets in Chinese Jujube by Small RNA and Degradome Sequencing
Source: Int J Mol Sci. 2026 Sep 4;27(17):7912. doi: 10.3390/ijms27177912 (PMC13566890; doi:10.3390/ijms27177912)
Supplement: Supplementary file 1 [file ijms-27-07912-s001.zip › Supplementary Material S2/novel_miR_122.pdf]

Provisional ID : novel_miR_122
Score total : 9286.5
Score for star read(s) : 3.9
Score for read counts : 9280.5
Score for mfe : 0.6
Score for randfold : 1.6
Score for cons. seed :
Total read count : 18215
Mature read count : 18165
Loop read count : 0
Star read count : 50

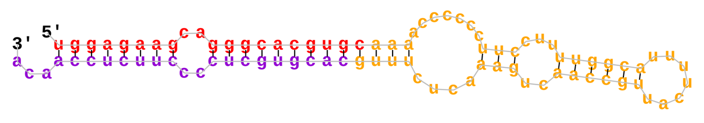

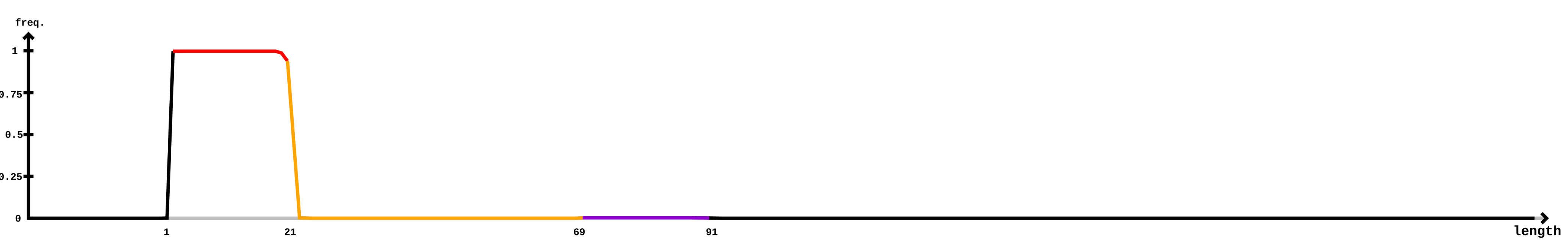

Mature Star

```
5'- uuuuugguguguagcuccuuguuggagaagcagggcacgugcaaaacccccccuuccuuuuggcauuuucauugccaacugaaacucuuugcacgugcuccccuucuccaacauggguuccucaccuuugucccucuauguuuaaccaccauuguauuauauauaaucuuagguaaugcaauaucauuacucuuuguuuuucuauauccucucugcuuucaguuucugauuaacauuauuuauuuauuuac  -3'   obs
    uuuuugguguguagcuccuuguuggagaagcagggcacgugcaaaacccccccuuccuuuuggcauuuucauugccaacugaaacucuuugcacgugcuccccuucuccaacauggguuccucaccuuugucccucuauguuuaaccaccauuguauuauauauaaucuuagguaaugcaauaucauuacucuuuguuuuucuauauccucucugcuuucaguuucugauuaacauuauuuauuuauuuac          exp
    .....((((.(.(((.((.((((((((((..((((((((((((.......(((...((((((.......))))))..)))....))))))))))))..))))))))))).))))).).))))...........(((((........((((((((((.((......)).))))))))))..................................((((...)))).)))))...............   reads   mm   sample
    .....................Cuggagaagcagggcacgu.........................................................................................................................................................................   1   1   LT-1
    .....................Cuggagaagcagggcacgugc.......................................................................................................................................................................   4   1   JCK-3
    .....................Cuggagaagcagggcacgugc.......................................................................................................................................................................   1   1   LT-2
    .....................uuggagaagcagggcacgugc.......................................................................................................................................................................   3   0   JCK-2
    .....................Cuggagaagcagggcacgugc.......................................................................................................................................................................   3   1   JCK-2
    .....................uuggagaagcagggcacgugc.......................................................................................................................................................................   1   0   LT-1
    .....................uuggagaagcagggcacgugc.......................................................................................................................................................................   1   0   JCK-3
    .....................uuggagaagcagggcacgugc.......................................................................................................................................................................   3   0   LT-3
    .....................Cuggagaagcagggcacgugca......................................................................................................................................................................   1   1   LT-1
    .....................Cuggagaagcagggcacgugca......................................................................................................................................................................   1   1   LT-3
    .....................Cuggagaagcagggcacgugca......................................................................................................................................................................   3   1   JCK-3
    ......................uggagaagcagggcacgu.........................................................................................................................................................................   19   0   LCK-3
    ......................uggagaagcagggcacgu.........................................................................................................................................................................   34   0   LT-3
    ......................uggagaagcagggcacgu.........................................................................................................................................................................   16   0   JCK-2
    ......................uggagaagcagggcacgC.........................................................................................................................................................................   1   1   LT-3
    ......................uggagaagcagggcacgu.........................................................................................................................................................................   2   0   JCK-1
    ......................uggagaagcCgggcacgu.........................................................................................................................................................................   1   1   JCK-2
    ......................uggagaagcagggcacgu.........................................................................................................................................................................   33   0   LT-1
    ......................uggagaagcagggcacgu.........................................................................................................................................................................   31   0   JT-2
    ......................uggagaagcagggcacgu.........................................................................................................................................................................   26   0   JT-3
    ......................uggagaagcagggcacgu.........................................................................................................................................................................   21   0   LT-2
    ......................uggagaagcagggcacgu.........................................................................................................................................................................   9   0   JCK-3
    ......................uggagaagcCgggcacgug........................................................................................................................................................................   1   1   LT-2
    ......................uggagaagcagggcacgug........................................................................................................................................................................   49   0   LCK-3
    ......................uggagaagcagggcacgug........................................................................................................................................................................   54   0   JT-2
    ......................uggagaagcagggcacguA........................................................................................................................................................................   7   1   LT-2
    ......................uggNgaagcagggcacgug........................................................................................................................................................................   1   1   JCK-1
    ......................ugAagaagcagggcacgug........................................................................................................................................................................   1   1   LT-2
    ......................uggagaagcagggcacgug........................................................................................................................................................................   41   0   JCK-3
    ......................uggagaagUagggcacgug........................................................................................................................................................................   1   1   LT-1
    ......................uggagaagcagggNacgug........................................................................................................................................................................   1   1   LCK-1
    ......................uggagaagcagggcacgug........................................................................................................................................................................   76   0   JT-3
    ......................uggagaagcagggcacgug........................................................................................................................................................................   53   0   JCK-2
```

Mature **Star**

```
uuuuugguguguagcuccuuguuggagaagcagggcacgugcaaaaccccccuuccuuuuggcauuuucauugccaacugaaacucuuugcacgugcucccuucuccaacauggguuccucaccuuugucccucuauguuuaaccaccauuguauuauauauaaucuuagguaaugcaauaucauuacucuuuguuuucuauauccucucugcuuucaguuucugauuaacauuauuuauuuauuuac
......................uggagCagcagggcacgug      1     1   LT-1
......................uggagaagcagggcaAgug      1     1   LT-2
......................uggagaagcagggcacgug      122   0   LCK-2
......................uggagaagcagggcacguA      1     1   LT-3
......................uggagaagcagggcacgug      37    0   JCK-1
......................uggagaagcagggcacgug      122   0   LT-3
......................uggagCagcagggcacgug      1     1   JT-3
......................uggagaagcagggcacguC      3     1   LT-3
......................uggCgaagcagggcacgug      1     1   LT-3
......................uggagaagUagggcacgug      1     1   JCK-2
......................uggagaagcaUggcacgug      1     1   JT-3
......................uggagaagcagggcacgug      127   0   LCK-1
......................uggCgaagcagggcacgug      1     1   LT-2
......................uggagaagcagggcacgug      66    0   LT-1
......................uggagaagcagggcacgug      109   0   LT-2
......................uggagaagcagggcacguA      2     1   JT-3
......................uAgagaagcagggcacgugc     1     1   JCK-3
......................uggagaagcagAgcacgugc     1     1   LT-3
......................uggagaagcagggNacgugc     1     1   LCK-1
......................uggagaCgcagggcacgugc     1     1   LCK-2
......................uggagaCgcagggcacgugc     1     1   JT-1
......................uggagaagcagggcacgugU     16    1   LT-3
......................uggagaagcCgggcacgugc     1     1   JCK-1
......................uggagaagcagggcNcgugc     2     1   LCK-1
......................Cggagaagcagggcacgugc     1     1   JCK-3
......................uggagaagcagggNacgugc     1     1   JT-2
......................uggNgaagcagggcacgugc     2     1   JT-2
......................uggagaCgcagggcacgugc     6     1   JCK-2
......................uggagaagcagggcacgugU     11    1   JCK-3
......................uggCgaagcagggcacgugc     11    1   LT-2
......................uNgagaagcagggcacgugc     2     1   JCK-2
......................uNgagaagcagggcacgugc     1     1   JCK-3
......................uggagaagcagggcCcgugc     3     1   JCK-1
......................uggagCagcagggcacgugc     4     1   LT-3
......................uggagaagcagggcacgugc     2130  0   LT-1
......................uggagaagcagggcaUgugc     2     1   JCK-2
......................uggagaagcagggcacgugc     1129  0   JCK-3
......................uggagaagcCgggcacgugc     1     1   JCK-3
......................uggagCagcagggcacgugc     2     1   JCK-2
......................uAgagaagcagggcacgugc     4     1   LT-2
......................uggagaagcagggcCcgugc     2     1   LT-2
......................uggCgaagcagggcacgugc     3     1   JCK-2
......................uggagaagcagggcacgugc     604   0   JT-2
......................uggagaagcagggcaUgugc     1     1   JCK-1
......................uggagaagcagggcacgugU     2     1   JT-3
......................uggagCagcagggcacgugc     3     1   LT-1
......................uggagaagcagggcacgugc     318   0   LCK-2
......................uggagaagcagggcacgugU     21    1   JCK-2
......................uggagaagcCgggcacgugc     5     1   LT-3
......................uNgagaagcagggcacgugc     1     1   LCK-1
......................Cggagaagcagggcacgugc     1     1   LT-3
......................uggagaagcagggcacgugc     532   0   LCK-3
......................Cggagaagcagggcacgugc     2     1   LT-2
......................uggagaagcagggcCcgugc     1     1   LCK-2
......................ugAagaagcagggcacgugc     2     1   LT-2
......................uNgagaagcagggcacgugc     1     1   LT-1
......................uggCgaagcagggcacgugc     1     1   LCK-1
......................uggagCagcagggcacgugc     1     1   JCK-3
......................uggagaagcCgggcacgugc     1     1   JCK-2
......................uAgagaagcagggcacgugc     1     1   JCK-1
......................uggCgaagcagggcacgugc     1     1   JCK-1
......................uggagaagcagggcacgugc     857   0   JT-3
......................uggagaCgcagggcacgugc     1     1   JT-2
......................uggagCagcagggcacgugc     2     1   JCK-1
......................uggagaagUagggcacgugc     10    1   JCK-2
......................uggagaagcCgggcacgugc     1     1   JT-2
......................uggagaCgcagggcacgugc     2     1   LT-1
......................uggagaagcagggcCcgugc     1     1   JT-3
......................uggagaCgcagggcacgugc     3     1   LT-2
......................uggagaagcagggcacgugU     11    1   LCK-3
```

Mature **Star**

uuuuggugugugagcuccuugu**uggagaagcagggcacgugc**aaaacccccuuccuuuuggcauuuucauugccaacugaaacucuuug**cacgugcuccccuucuccaaca**uggguuccucaccuuugucccucuauguuuaaccaccauuguauuauauauaaucuuagguaaugcaauaucauuacucuuuguuuuucuauaucccucucugcuuucaguuucugauuaacauuauuuauuuauuuac

uggagaCgcagggcacgugc	3	1	JCK-3
uggaAaagcagggcacgugc	1	1	LT-3
uggagaagcagggcacgugU	25	1	LT-1
uggagaCgcagggcacgugc	7	1	LT-3
uggagaagUagggcacgugc	3	1	LT-3
uggagaagcagggcacgCgc	1	1	LT-3
uggCgaagcagggcacgugc	4	1	LT-3
uggagaagcagggcCcgugc	3	1	LT-3
uggagaagcCgggcacgugc	1	1	LCK-3
uggagaagUagggcacgugc	3	1	LT-2
ugAagaagcagggcacgugc	1	1	LCK-3
uggagGagcagggcacgugc	1	1	LCK-1
uggagaagcagggcacgugc	319	0	LCK-1
uggagaagcagggcCcgugc	3	1	JCK-2
uggCgaagcagggcacgugc	1	1	LT-1
uggagaagcagggcCcgugc	1	1	JT-2
uUgagaagcagggcacgugc	1	1	JCK-3
uggagaagcCgggcacgugc	1	1	LT-1
uggagaagcagggUacgugc	1	1	JCK-3
uggagaagcagNgcacgugc	2	1	JCK-1
Gggagaagcagggcacgugc	1	1	LCK-2
uggagaagcagggcacgugN	1	1	JCK-1
uggagaagAagggcacgugc	1	1	JCK-2
uggagaagcagggcacgugc	1369	0	LT-2
uggagaagcagggcacgugc	2380	0	LT-3
uggagaagcagggcCcgugc	1	1	LT-1
uggagaagcagggcaUgugc	2	1	LT-3
uggagaagUagggcacgugc	1	1	LT-1
uggagaagcagggcGcgugc	1	1	LCK-1
uggagaagcagggcacgugU	44	1	LT-2
uggagaagcCgggcacgugc	3	1	LT-2
uAgagaagcagggcacgugc	1	1	LT-1
uggagaagcagggcacguAc	1	1	JCK-2
uggagaagcagggcacgugc	121	0	JT-1
uggaAaagcagggcacgugc	1	1	JT-3
ugNagaagcagggcacgugc	3	1	LT-2
uggagaagcagggcCcgugc	1	1	JCK-3
uggagCagcagggcacgugc	1	1	LT-2
uggagaagcaggAcacgugc	1	1	LCK-3
uggagGagcagggcacgugc	1	1	LT-1
ugNagaagcagggcacgugc	1	1	LCK-1
ugAagaagcagggcacgugc	1	1	JCK-3
uggGgaagcagggcacgugc	1	1	LCK-3
uggCgaagcagggcacgugc	1	1	JCK-3
uggagaagcagggcacgugc	875	0	JCK-1
uggagaagcGggcacgugc	2	1	LT-3
uggagaagUagggcacgugc	7	1	JCK-3
uggagaagcagggcacgugc	1625	0	JCK-2
uggNgaagcagggcacgugc	1	1	JCK-1
uggagaagUagggcacgugc	2	1	JCK-1
uggagaagUagggcacgugc	2	1	JT-3
uggagaagcagggcacgugU	4	1	JCK-1
uggCgaagcagggcacgugc	1	1	JT-2
uggagaagcagggcacgugcU	10	1	LT-2
uggCgaagcagggcacgugca	1	1	JCK-1
uggagaagcagggcacgugca	92	0	JT-2
uggagaagcagggcacgugcC	2	1	JCK-1
uggagaagcagggcacgugca	360	0	LT-1
uggagaagcagggcacgugca	317	0	LT-3
uggagaagcagggcacgugUa	1	1	LT-1
uggagaagcagNgcacgugca	2	1	JCK-1
uAgagaagcagggcacgugca	1	1	JCK-3
uggagaagcagggcacAugca	2	1	JT-2
uggagaagcagggcaGgugca	1	1	JCK-2
uggagaagcagggcacgugcC	9	1	JCK-2
uggNgaagcagggcacgugca	1	1	JCK-1
uggagaagcagggcaAgugca	1	1	JCK-1
uggCgaagcagggcacgugca	2	1	LT-1
uggagaagcagggcacgugcG	1	1	JCK-2
uggagaagcagggcacgugca	100	0	JT-1

```
                      Mature                                                                   Star
uuuuggguguguagcuccuuguuggagaagcagggcacgugcaaaacccccuuccuuuuggcauuuucauugccaacugaaacucuuugcacgugcuccccuucuccaacaugggguuccucaccuuugucccucuauguuuaaccaccauuguauuauauauaaucuuagguaaugcaauaucauuacucuuuguuuuucuauauccucucugcuuucaguuucugauuaacauuauuuauuuauuuac
......................uggagaagcagggcacgugcU     1     1    JCK-1
......................uggagaagcagggcacgugca     58    0    LCK-3
......................uggagaagcagggcacgugcU     19    1    LCK-3
......................uggagaagcagggcacgugcC     3     1    LT-3
......................uNgagaagcagggcacgugca     1     1    JCK-3
......................uggagaagcagggcacgugcC     4     1    JCK-3
......................uggagaagcagggcacAugca     1     1    JT-3
......................uggagaagcagggcCcgugca     1     1    JCK-3
......................uNgagaagcagggcacgugca     1     1    JCK-1
......................uggagaagcagggcacgugNa     1     1    JCK-1
......................uggagaagcagggcacgugca     805   0    JCK-1
......................uggagaagcagggcacgugcC     4     1    LT-2
......................uggagCagcagggcacgugca     1     1    JCK-3
......................uggagaCgcagggcacgugca     1     1    JCK-1
......................uggagCagcagggcacgugca     1     1    JCK-1
......................uggagaagUagggcacgugca     1     1    LT-1
......................uggagaagcagggcacgugca     1285  0    JCK-2
......................uggagaagcagggcacgugca     2     0    LCK-2
......................uggagaagUagggcacgugca     6     1    JCK-2
......................uggagaagUagggcacgugca     6     1    JCK-3
......................uggagaagcagggcCcgugca     1     1    JCK-2
......................uggagaagcCgggcacgugca     1     1    JCK-1
......................uggagaCgcagggcacgugca     1     1    JCK-3
......................uggagaagUagggcacgugca     1     1    JT-1
......................uggagaagcagggcaUgugca     1     1    JCK-2
......................uggagaagcagggcacgugca     131   0    JT-3
......................uggagaagcagggcacgugcU     7     1    JCK-2
......................uggagaCgcagggcacgugca     3     1    JCK-2
......................uggagCagcagggcacgugca     1     1    LT-2
......................uggagaagcagggcaGgugca     1     1    JCK-1
......................uggagaagcagggcCcgugca     1     1    LT-1
......................uggagaagcagggcCcgugca     1     1    LT-2
......................uNgagaagcagggcacgugca     5     1    JCK-2
......................uggagaagcagggcacgugcU     13    1    LT-1
......................uggagaagcagggcacgugUa     1     1    LT-3
......................uggagaagcagggcacgugcC     7     1    LT-1
......................uggagaCgcagggcacgugca     1     1    LT-3
......................uggagaagcagggcacgugca     143   0    LT-2
......................uggagaagcagggcacgugca     1008  0    JCK-3
......................uggagaagUagggcacgugca     3     1    JCK-1
......................uggagaCgcagggcacgugca     1     1    JT-3
......................uggagaagcagggcacgugUa     4     1    LT-2
......................uggagaagcagggcGcgugca     1     1    JCK-3
......................uggagaagcagggcacgugcU     6     1    LT-3
......................uggagaagcagggUacgugca     1     1    LT-1
......................uggagaagcagggcacgugcU     16    1    JT-1
......................uggagCagcagggcacgugca     1     1    JCK-2
......................uggCgaagcagggcacgugca     1     1    JCK-2
......................uggagaagcagggcacgugcaC    1     1    JCK-1
......................uggagaagcagggcacgugcaa    1     0    JCK-1
......................uggagaagcagggcacgugcaa    3     0    JCK-3
......................uggagaagcagggcacgugcaG    1     1    LT-1
......................uggagaagcagggcacgugcaa    3     0    JCK-2
......................uggagaagcagggcacgugcaa    1     0    LT-1
......................uggagaagcagggcacgugcaCa   2     1    LT-1
......................uggagaagcagggcacgugcaaa   21    0    JT-1
......................uggagaagcagggcacgugcaaa   1     0    LT-1
........................gagaagcagggcacgugc      2     0    JCK-3
.........................................................................................cacgugcuccccuucucc        1     0    JCK-1
.........................................................................................cacgugcuccccuucucca       3     0    JCK-2
.........................................................................................cacgugcuccccuucucca       2     0    JCK-1
.........................................................................................cacgugcuccccuucucca       6     0    LCK-3
.........................................................................................cacgugcuccccuucuccaa      2     0    LT-3
.........................................................................................cacgugcuccccuucuccaac     2     0    LT-1
.........................................................................................cacgugcuccccuucuccaac     3     0    JCK-3
.........................................................................................cacgugcuccccuucuccaac     2     0    JCK-1
.........................................................................................cacgugcuccccuucuccaac     5     0    JCK-2
.........................................................................................cacgugcuccccuucuccaaA     1     1    LT-1
.........................................................................................cacgugcucUccuucuccaac     1     1    LT-3
.........................................................................................cacgugcuccccuucuccaacU    3     1    LT-2
```

```
                  Mature                                                                   Star
uuuuugguguguagcuccuuguuggagaagcagggcacgugcaaaacccccccuuccuuuuggcauuuucauugccaacugaaacucuuugcacgugcuccccuucuccaacauggguuccucaccuuugucccucuauguuuaaccaccauuguauuauauauaaucuuagguaaugcaauaucauuacucuuuguuuuucuauauccucucugcuuucaguuucugauuaacauuauuuauuuauuuac
...........................................................................................cacgugcuccccuucuccaacU..........................................................................................................................................	5	1	JCK-1
...........................................................................................cacgugcuccccuucuccaacU..........................................................................................................................................	3	1	LT-1
...........................................................................................cacgugcuccccuucuccaacU..........................................................................................................................................	2	1	JCK-3
...........................................................................................cacgugcuccccuucuccaacU..........................................................................................................................................	8	1	JCK-2
............................................................................................acgugcuccccuucucca.............................................................................................................................................	1	0	JCK-2
```
